# Cardiac Implantable Electronic Devices and Consumer Electronic Devices: The Proof Is in the Front Pocket

**DOI:** 10.19102/icrm.2022.130706

**Published:** 2022-07-15

**Authors:** Christopher R. Ellis, Nicholas E. King

**Affiliations:** ^1^Vanderbilt University Medical Center, Nashville, TN, USA

**Keywords:** Cell phone, defibrillator, implantable cardioverter-defibrillator, magnet

Patel et al.^1^ ask a question whose answer is key in the modern age of telecommunication: do current-generation cellular devices result in cardiac implantable electronic device (CIED) malfunction? Their observational study with the specific concern about the array of magnets involved in wireless inductive charging showed that short-interval, close-proximity exposure of multiple implantable cardioverter-defibrillators (ICDs) to an iPhone 12 resulted in no effect. However, both cellular devices and CIEDs are complex technological devices, and interpreting this study requires additional context.

Initially, CIEDs were programmed by physical manipulation of the device via percutaneous access, which, given the infection and complication risks, spurred the development of non-invasive programming options. The earliest design used static external magnetic fields to either adjust magnetic knobs within the CIED or initiate a cycling of pacing options by engaging a reed switch, a mechanical switch that engages when exposed to a magnetic field. As CIED programming progressed, initial non-invasive programmers used the principle of electromagnetic induction to transmit programming instructions via pulsed magnetic fields, resulting in the opening and closing of an internal CIED reed switch. The pattern of switching on and off was decoded by the device and then translated into a programming change. However, as the reed switch was a magnetically activated mechanical switch, it was prone to reprogramming from “chattering” due to non-intentional magnetic exposure and simple mechanical failure.

The mechanical failure of the reed switch was bypassed with the advent of magnetic coupling via an internal CIED coil; however, the inadvertent exposure of the coil to magnetic fields remained. To eliminate magnetic interference, CIED programming was transitioned to radiofrequency (RF) transmission, with modern programmers encoding complex instructions via pulsed electromagnetic waves across a spectrum of frequencies to limit the effects of magnetic interference.^2^

The RF spectrum (3 Hz–3 THz) is regulated by the Federal Communications Commission in the United States, with medical device communication band allocations of 175 kHz and 402–405 MHz.^3,4^ However, significant electromagnetic interference (EMI) can occur due to electromagnetic fields or movement through a sufficiently powerful static magnetic field.^5^ Modern CIEDs use advanced programming features such as access codes to initiate the CIED’s programming mode and bidirectional RF transmission to allow for device–programmer validation.^2^ They also incorporate design features, such as material shielding of the generator and leads, to reduce EMI susceptibility.^5^ Bipolar lead sensing with low-pass or feed-through filters reduces radiated interference, bypass filters eliminate frequencies outside the cardiac range, and noise reversion programming all help to limit EMI.^5^ Despite these safeguards, EMI in the proper frequency and/or strength can cause CIED malfunction, and exposure to everyday devices like washing machines, power tools, and electronic security systems may rarely result in CIED malfunctions typically due to the abnormal bipolar sensing of RF EMI in the programmed range of interest.^5^

Exposure to industrial levels of EMI with large current applications, such as arc welding, high electric currents, industrial electric motors, and magnetic coils, is not recommended; however, documented cases of significant EMI without contact with the electrical source are rare.^5–7^ Ironically, the largest risk of clinically significant EMI exposure is within health care from magnetic resonance imaging (MRI), electrosurgery procedures such as RF ablation, and ionizing electromagnetic radiation typically in the form of therapeutic radiation.^5,8–12^ The most feared complication of EMI is a “power-on reset,” where the CIED experiences such a significant EMI event that its programmable functions are corrupted and the device resets to a back-up read-only program (typically DDI or VVI), typically with a loss of critical pacing or ICD function.

CIEDs still use magnetic sensors, although no longer via mechanical reed sensors. In a sufficiently strong magnetic field, current-generation sensors such as the giant magneto-sensitive resistors generate a change in resistance, Hall sensors detect a change in voltage, and telemetry coils detect a change in current to serve as the detection signal—the specifics are brand-dependent.^13^ While a magnetic strength of 10 G (the strength of a standard refrigerator magnet) can induce EMI, a strength of ≥90 G is typically required to switch pacemakers to an asynchronous pacing mode or to disable ICD therapies (magnet inhibition).^5,13–15^

MRIs represent a uniquely challenging operational space for CIEDs as they emit both a fixed magnetic field on the order of teslas (1 T = 10,000 G) and RF radiation for tissue excitation. Older CIEDs that use reed sensors are especially prone to malfunction due to their unpredictable behavior in the setting of multimodal EMI.^13,16,17^ Modern MRI conditional devices incorporate fewer ferromagnetic elements and better internal EMI shielding; some even incorporate “MRI mode” programming, which pre-emptively changes some elements of the CIED function.^5,17,18^ However, even non–MRI-conditional devices can undergo MRI with a minimal risk of adverse CIED function if the device was implanted in roughly the year 2000 or later due to the inherent changes in design such as the elimination of reed sensors.^19,20^

Given the rise of wearable communication technology, there has been growing concern regarding the potential impact of EMI from consumer cellular devices on CIEDs. Cellular devices produce numerous signals, including cellular data and Bluetooth^®^ RF signals ranging from 700–2,700 MHz.^21,22^ Testing of EMI from an iPhone 6 and Apple Watch A1553 (Apple, Cupertino, CA, USA) against 148 patients with CIEDs showed that only the iPhone when placed directly over the CIED caused 14% of patients to experience EMI (defined as a change in cardiac rhythm on surface electrocardiogram with or without symptoms), with no evidence of a magnet effect.^23^ More recently, inductive loops have been incorporated into a range of devices to allow for wireless charging based on Faraday’s law of electromagnetic induction; significant concern has been raised regarding the potential of EMI from these inductive loops as they can produce significant EMI in the form of fluxing magnetic fields.

The magnetic strength of the Qi A13 wireless charging board (Samsung, Seoul, South Korea) was experimentally measured and found to be up to 1.27 G during the pre-charging “pinging” mode 2 cm from the board surface, which rapidly decayed to 0.024 G at 10 cm (units converted to G), well below the 90-G threshold.^24^ Additionally, fixed rare-earth magnets incorporating elements such as neodymium, which are significantly stronger than the more common ferrite magnets, are being broadly incorporated into devices to provide attachment stability. Measurements collected from the AirPods Pro and their wireless charging case (Apple), the Microsoft Surface Pen (Microsoft Corporation, Redmond, WA, USA), and the second-generation Apple Pencil show that they produce magnetic fields of 10 G at distances as far as 29 mm, which are sufficient enough to cause EMI (peak magnetic fields are not reported).^25^ Measurements of the Apple Watch body report magnetic fields of 983 G at 1 mm, which attenuates to 39 G at 11 mm.^26^ Case reports of reproducible magnet inhibition of MRI-compatible ICDs (e.g., Visia AF MRI S DF-1 single-chamber ICD; Medtronic, Minneapolis, MN, USA) with a patient’s Apple Watch wristband magnet and another (Medtronic Evera MRI XT DR DDMB1D1) with the magnetic charging components of an electronic cigarette vaping device (JUUL, San Francisco, CA, USA) highlight the need for further investigation into personal consumer electronics and CIED EMI.^27,28^

Specifically, Apple has developed a MagSafe charging technology, which is present in the iPhone versions 12 and 13 and expected to be included in all future devices. It consists of an inductive charging coil surrounded by a fixed neodymium magnetic ring in the cellular device that mates to a set of opposite polarity magnets in the inductive charging puck. Magnet measurements of the iPhone 12 show that it produces magnetic fields of up to 190 G at 1 mm and up to 19 G at 11 mm.^26^ A single patient report showed that bringing an iPhone 12 in close proximity to a Medtronic ICD (device not specified) resulted in magnet inhibition.^29^ Another case series involving positioning an iPhone 12 over both non–MRI-compatible and MRI-compatible ICD and pacemaker devices from a variety of manufacturers in vivo (3 patients) and ex vivo (11 devices) showed that 100% and 72.7% of devices had magnet inhibition, respectively.^30^ A larger study similarly found a CIED interference rate of up to 84.6% with the back of the iPhone 12 facing the CIED across 36 ex vivo devices but a rate of only 18.3% for in vivo devices across 164 patients, again with the back of the phone facing the CIED, leading to the study’s recommendation to “flip it [the phone].” Notably, the magnetic activation distance of the iPhone 12 when advanced toward the CIED was significantly lower than that of the clinical magnet (average of 3.6 mm vs. minimum of 4 cm). Magnetic field measurements revealed iPhone magnetic field strengths of up to 204 G at 1 mm.^31^ These findings stand in contrast to the present study involving 17 patients with CIEDs who reported no interference when exposed to the iPhone 12; another study considering the Apple iPhone 12 Pro Max, Apple iPhone XR, Samsung Galaxy S21, and Samsung Galaxy S8 also reported no magnet inhibition in 12 patients with CIEDs.^32^

Currently, the U.S. Food and Drug Administration recommends a minimum safe distance of 15 cm between CIEDs and consumer electronics with magnetic components.^33^ Older CIEDs (implanted before 2000) are the highest-risk devices due to their inherent lack of modern safety EMI countermeasures and prevalence of mechanical reed switches. However, even modern CIEDs (both non–MRI- and MRI-compatible) show clear evidence of potential EMI from current consumer devices. Static measurements show that, at sufficiently closer distances, consumer devices generate magnetic fields capable of activating a CIED’s magnet inhibition.^26,31^ This has also clearly been shown in stand-alone CIEDs and implanted devices.^27–31^ However, given the proliferation of these devices (>100 million units of the iPhone 12 alone), it is reassuring that, to date, only case reports of consumer device–induced magnet inhibition with real-world use have been published.^27,28^ Additionally, even in experimental settings where the device is intentionally placed in proximity to the CIED, only a small portion of the study population experienced magnet inhibition or interference, which would be expected to resolve once the device was moved.^23,31^

The current study by Patel et al.^1^ and the previous one by Held et al.^32^ were unable to show any EMI. No episodes of power-on resets induced by consumer devices have been reported, nor would they be expected with the measured levels of EMI. Therefore, while there is a distinct risk of EMI and potential magnet inhibition by current consumer electronic devices, the current risk seems to be overstated. A minimum distance of 2 cm would likely be adequate to sufficiently decay the magnetic field strength to subclinical levels.^25,26,31^ Also, while directions to patients should be clear about this distance, evidence suggests that, even if it was exceeded, the risk of EMI or magnet inhibition is low. Thus, while patients should be counseled not to place electronic devices in the shirt pocket overlying their CIED, the occasional lapse is unlikely to result in a clinically meaningful CIED malfunction.

However, as consumer electronics and environment-level EMI proliferate (e.g., potential in-ground magnetic induction loops for electronic vehicles), ongoing work is needed to ensure the compatibility of CIEDs. Device manufacturers should continue to incorporate additional EMI shielding technology into base CIED designs, not just MRI-compatible devices, as the general risk in the out-of-hospital environment increases. The International Commission on Non-ionizing Radiation Protection guidelines limit environmental magnetic field exposure to <0.8 G, which is significantly lower than the CIED interference threshold, but clinicians should still remain vigilant for any potential unanticipated effects.^34^
